# The Mechanism of Celosiae Semen in the Treatment of Diabetic Cataract: Based on Network Pharmacology

**DOI:** 10.2174/0118715303387305250818132910

**Published:** 2025-09-11

**Authors:** Song Caihong, Hao Fang, Hui Song

**Affiliations:** 1 Tianjin Key Laboratory of Retinal Functions and Diseases, Tianjin Branch of National Clinical Research Center for Ocular Disease, Eye Institute and School of Optometry, Tianjin Medical University Eye Hospital, Tianjin, China;; 2 Department of Cardiothoracic Surgery, Tianjin Medical University General Hospital, Tianjin, China

**Keywords:** Celosiae semen, diabetic cataract, network pharmacology, molecular docking, molecular dynamics simulation, ocular diseases

## Abstract

**Introduction:**

Diabetes mellitus can be complicated by a variety of ocular diseases, among which the postoperative complications of diabetic cataract (DC) are significantly higher than those of non-DC patients. Therefore, finding drugs with natural active ingredients is an urgent challenge in the prevention and treatment of DC. Discovering the potential molecular mechanism of celosiae semen (CS) for the treatment of DC and providing new ideas and programs for the treatment and prevention of DC.

**Methods:**

In this study, network pharmacology, molecular docking, and molecular dynamics simulations were utilized to predict the binding and functional enrichment of the main active ingredients of CS with DC-related targets, and to explore the potential pathways and mechanisms of CS for the treatment of DC.

**Results:**

Through database searching and screening, a total of 45 potential targets of CS for the treatment of DC were identified, functionally enriched, and a protein-protein interaction network was constructed, and the key target, SRC, was finally found. The results of molecular docking and molecular dynamics simulation showed that the main active ingredient of CS, stigmasterol, could bind stably to the key target SRC protein.

**Discussion:**

This study not only elucidates the phyto-pharmacological basis of CS in DC management but also provides a framework for developing natural product-derived targeted therapies against diabetic ocular complications. The integration of modern genomics and computational chemistry to deconstruct the therapeutic effects of traditional Chinese herbal medicines has great clinical significance in expanding the scope of traditional Chinese medicines for the treatment of DC and promoting precision targeting. However, this requires verification through basic experiments.

**Conclusion:**

These computational findings suggest that CS may exert its anti-cataract effects through the multi-target modulation of diabetic metabolic pathways and SRC-mediated signaling cascades.

## 1 INTRODUCTION

The incidence of diabetes is on the rise globally, and it is expected that the number of people with diabetes will rise to 629 million by 2045 [1]. The abnormal nutritional and metabolic state of diabetic patients can cause irreversible damage to many organs throughout the body, and its complications in the eye are the main cause of blindness [2-5]. Among them, the development and progression of diabetic cataract (DC) can severely interfere with retinal disease follow-up and intervention, further exacerbating the progression of retinopathy, which can lead to irreversible damage to the patient's vision [3, 6-8]. Some studies suggest that patients with DC will bear a higher risk of eye surgery [9, 10]. As for the unique ocular microenvironment of diabetic patients [11-15], the surgical risks of DC should still not be ignored, such as postoperative macular cystoid edema and vitreous hemorrhage, as well as accelerating the progression of diabetic retinopathy [11, 16-20], which will affect the patient's experience of the medical care and increase the additional consumption of healthcare resources [21-23]. Therefore, finding and developing drugs to prevent or treat crystalline clouding in patients with DC with safe and effective compositions is a challenge that remains to be solved.

As a natural herbal medicine with various effects such as protecting the liver, brightening the eyes, and reducing cataracts, celosiae semen (CS) has been reported to be rich in amino acids and trace elements, which can have a certain therapeutic effect on blurred vision and cataracts [24, 25]. CS has a variety of effects, such as liver protection, lowering blood sugar, anti-oxidative damage to the lens, and so on [25-27]. A study has shown that taking a decoction of CS can significantly improve the clouding of the crystalline lens in patients with age-related cataracts, thus improving their visual acuity [28]. Hamzah *et al.* verified that CS extract can reduce blood glucose levels and improve antioxidant capacity in diabetic rats [8]. Therefore, CS shows some potential in lowering blood glucose and treating cataracts, but its specific efficacy and mechanism in treating DC need to be further studied.

Network pharmacology efficiently analyzes the active ingredients and targets of a drug and combines them with the therapeutic sites of a disease to predict the potential targets of a drug's action on a disease [29, 30]. The perfect combination of molecular docking and molecular dynamics (MD) simulation can predict and evaluate the binding sites, binding energies, and docking configurations between active ingredients and disease targets, and thus accurately understand the mechanism of drug action and therapeutic potential [31-33]. Therefore, this study combined natural Chinese herbs with modern medical treatment, and applied several techniques, such as network pharmacology, molecular docking, and MD simulation, to predict the interactions between the active ingredients of CS and the relevant targets of DC, and to excavate the targets of CS’s action and therapeutic mechanism on DC.

## 2 METHODS

### 2.1 Acquisition of CS Active Ingredient Targets

The TCMSP database (https://old.tcmsp-e.com/tcmsp.php/) [34] was utilized to retrieve the active ingredients and their corresponding targets in CS, with bioavailability (OB) >30% and drug-like properties (DL) >0.18 as qualifiers. The target name was harmonized by the Uniprotdatabase (https://www.uniprot.org/) [35] and the GeneCards database (https://www.genecards.org/) [36], and the corresponding gene names were obtained by selecting human species sources.

### 2.2 Acquisition of DC-Related Druggable Targets

In this study, the GeneCards database, DisGeNET database (https://www.disgenet.org/) [37], and OMIM database (https://omim.org/) were searched with the keyword “diabetic cataract” and were combined with existing targets reported in the study [38]. All the DC-related druggable targets were obtained after removing duplicates in the GeneCards database using a relevance score >12 as the screening criterion.

### 2.3 Obtaining Potential Targets for CS Treatment of DC and Identifying Key Targets

Take the intersection of DC targets and CS druggable targets to obtain potential targets for CS to treat DC. Protein-protein interaction (PPI) networks were constructed in the STRING database (https://cn.string-db.org/) and imported into Cytoscape software, analyzed by the “cytoHubba” algorithm to obtain degree value information, and ranked. thereby identifying the key targets [39, 40].

### 2.4 Functional Enrichment Analysis

This study enriched the functions or potential pathways of potential targets of CS on DC to predict the underlying mechanisms using Gene Ontology (GO), Kyoto Encyclopedia of the Genome (KEGG), and Reactome [41].

### 2.5 Molecular Docking

The structures of the main target proteins were obtained from the AlphaFold protein structure database (https://alphafold.com/), and the structures of the active ingredients corresponding to these proteins were obtained from the PubChem database (https://pubchem.ncbi.nlm.nih.gov/) [42]. The proteins were dehydrogenated in Autodock Tools 1.5.7 software, followed by molecular docking in Autodock vina 1.2.5 software to predict the sites [43], conformations and binding energies of the proteins for binding to the drugs, and further visualized in Pymol 3.0.4 software.

### 2.6 MD Simulation

MD simulations were performed using the Gromacs2022 program with the GAFF force field for small molecules, the AMBER14SB force field for proteins, and the TIP3P water model to merge the files of proteins and small-molecule ligands to construct a simulation system for the complexes. This was carried out at constant temperature and pressure as well as under periodic boundary conditions. During the MD simulations, all hydrogen bonds involved were constrained using the LINCS algorithm with an integration step of 2 fs. Electrostatic interactions were calculated using the Particle-mesh Ewald (PME) method with a cutoff value of 1.2 nm, and non-bonded interactions with a cutoff value of 10 Å, which was updated every 10 steps. The simulation temperature was controlled by the V-rescale temperature coupling method at 298 K, and the pressure was controlled by the Berendsen method at 1 bar. 100 ps of NVT and NPT equilibrium simulations were performed at 298 K. 100 ns of MD simulations were performed for the complex system, and the conformation was saved every 10 ps. After completion of the simulations, the trajectories were analyzed using VMD and pymol.

### 2.7 Statistical Analysis

Statistical analyses were conducted using R software version 4.2.1. Pathway enrichment analysis was executed using the “clusterProfiler” package [44, 45], respectively. A significance level of *p* < 0.05 was considered statistically significant.

## 3 RESULTS

### 3.1 Identification of Key Targets and Construction of Interaction Networks for the Treatment of DC By CS

**Fig. (1):**
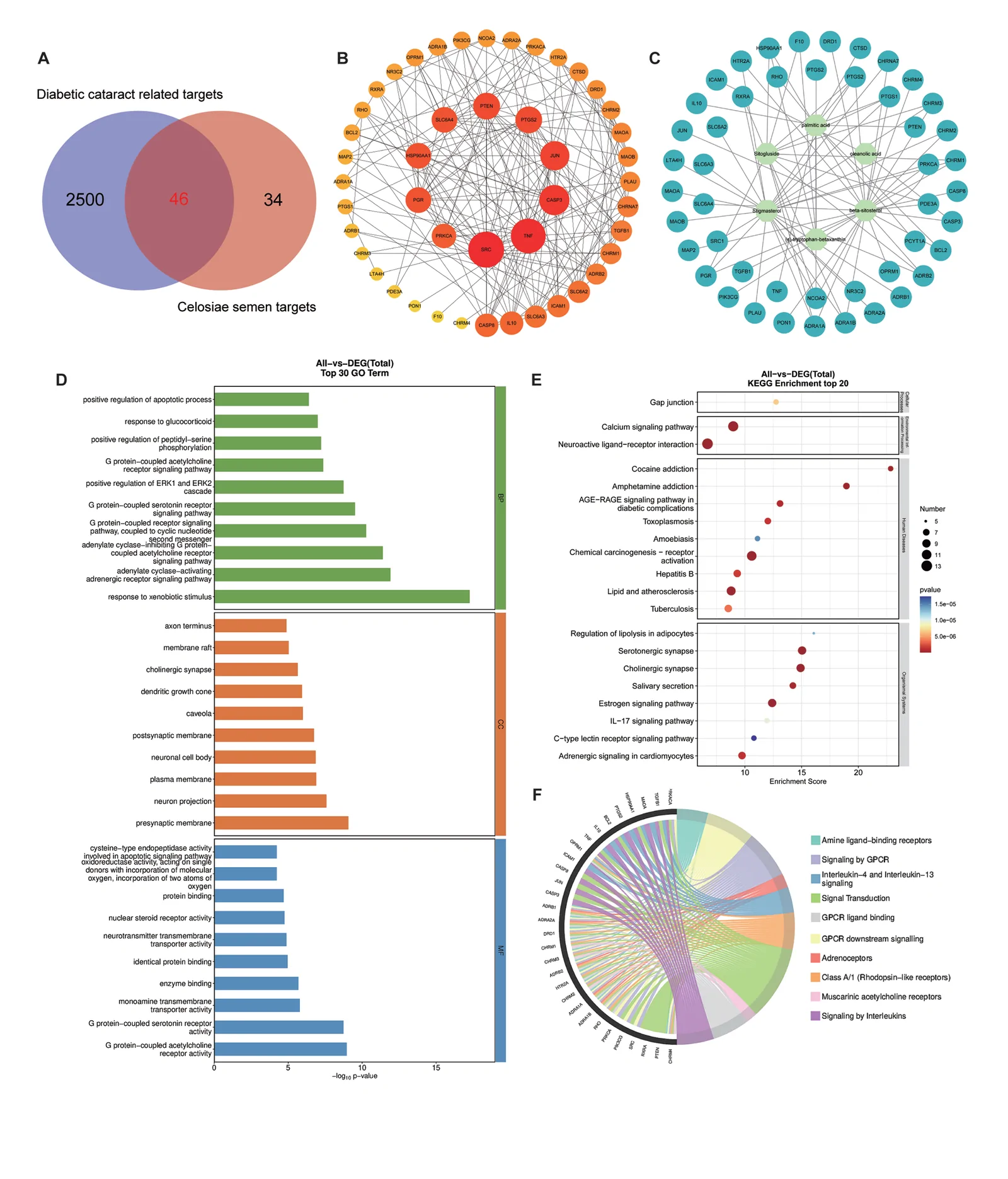
Key target screening and enrichment analysis. (**A**) Venn diagram of predicted celosiae semen targets and diabetic cataract related targets. (**B**) PPI network of intersecting targets. (**C**) Celosiae semen–targets network diagram. (**D**) GO enrichment analysis. (**E**) KEGG enrichment analysis. (**F**) Reactome enrichment analysis. GO, gene ontology; KEGG, Kyoko Encyclopaedia of Genes and Genomes.

A total of 6 active ingredients and 80 targets of CS were obtained, as well as 2546 DC-related targets. By calculating the intersection of CS targets with DC-related targets, 46 potential targets for CS treatment of DC were obtained. (Fig. **1A**). The active ingredients corresponding to these targets were selected to construct an active ingredient-target interaction network related to DC for CS treatment (Fig. **1B**), and the “cytoHubba” algorithm was utilized to identify the most central target SRC in the PPI network, and the degree value of TNF was relatively close to that of SRC, which was ranked as the second (Fig. **1C**).

### 3.2 Functional Enrichment Analysis

To explore the possible functions and downstream pathways played by these genes, enrichment analysis of intersecting targets was performed in this study. The 46 target genes for CS treatment of DC are mainly enriched in the KEGG database for 5-hydroxytryptaminergic synapses, cholinergic synapses, and AGE-RAGE signaling pathways (Fig. **1D**). And in the GO database for G protein-coupled receptor signaling pathways at the level of biological process (BP), and synaptic membranes and neuronal cell bodies at the level of cellular component (CC). The molecular function (MF) level is mainly enriched with pathways such as G protein-coupled correlative receptors and neurotransmitter transmembrane transport (Fig. **1E**). The Reactome database is mainly enriched in pathways related to GPCR, interleukins, *etc* (Fig. **1F**).

The structural simplicity of the six active components in the interaction network of CS is presented in Fig. (**2**).

### 3.3 Molecular Docking

**Fig. (3):**
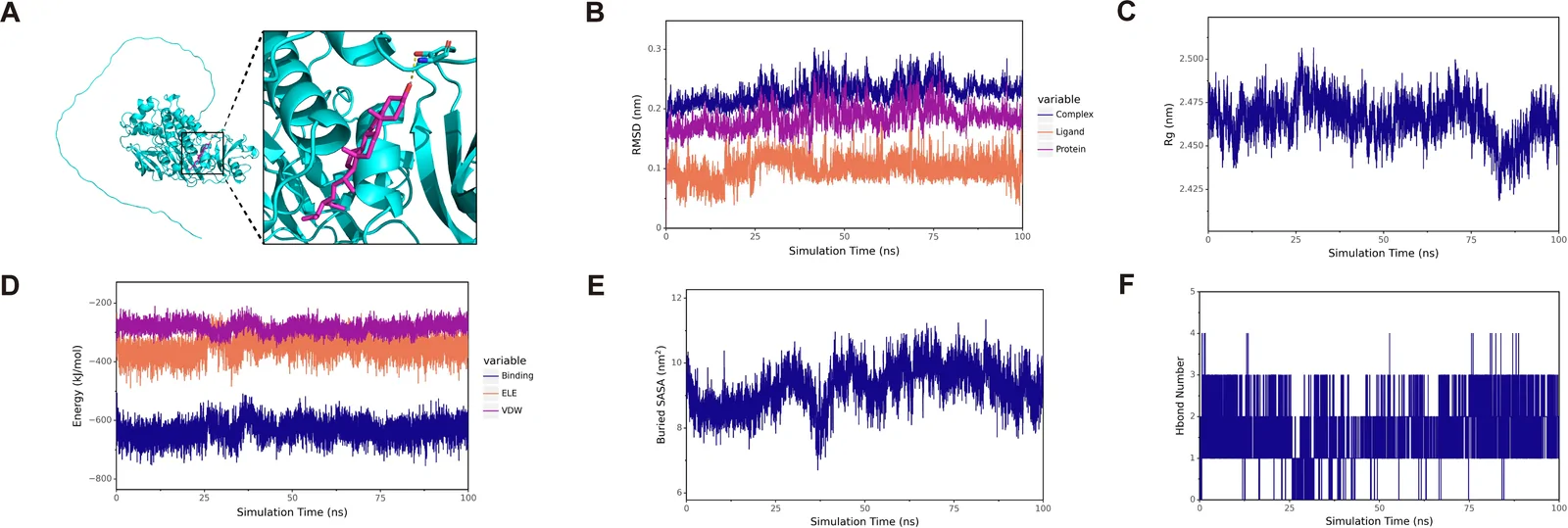
Results of molecular docking. (**A**) Interaction diagram between SRC and stigmasterol. (**B**) RMSD values between SRC and stigmasterol. (**C**) Radius of gyration (Rg) values between SRC and stigmasterol. (**D**) Binding energies between SRC and stigmasterol. (**E**) Solvent-accessible surface area (SASA) values between SRC and stigmasterol. (**F**) Number of hydrogen bonds between SRC and stigmasterol.

In order to assess the binding between the target protein and the active ingredient, the binding affinities of the first two key target genes (SRC and TNF) and their corresponding active ingredients were evaluated separately using the molecular docking technique. The results showed that the docking scores of these SRC and stigmastersol were -8.9 kcal / mol and were able to form stable hydrogen bonds (Fig. **3A**), whereas the docking scores of TNF and palmitic acid were only -4.1 kcal / mol. Lower docking scores indicate stronger binding affinity; scores less than -5.0 kcal / mol indicate potential binding, and scores less than -7.0 kcal / mol indicate stronger binding affinity.

### 3.4 MD Simulation

To further explore the stability of protein-ligand interactions, this study performed MD simulations of each of the SRC proteins with stigmasterol. MD simulations contain a number of parameters, the more important of which are included Root Mean Square Deviation (RMSD), Radius of Gyration (Rg), van der Waals, electrostatic interactions, and buried Solvent Accessible Surface Area (SASA), and by which the present study comprehensively assesses the binding status of SRC and stigmasterol. RMSD is a commonly used index to measure the difference between two structures. It reflects the similarity of the conformations of two molecules by comparing the spatial coordinates of the corresponding atoms of the two molecules, and is used to track the changes of the molecular structure during the simulation process with respect to the initial structure, and to observe whether the change amplitude tends to be stabilized or not. The lower the RMSD, the closer the two structures are. The RMSD of the complex structure and the RMSD of the protein gradually stabilized with the simulation, indicating that the complex structure was gradually stabilized (Fig. **3B**). Rg is the root-mean-square distance of all atoms in a molecule relative to the center of mass, reflecting the distribution of atoms in a molecule relative to the center of mass, and is an important parameter used to measure the degree of compactness of the overall structure of proteins and small molecules. The smaller the radius of gyration Rg is, the more compact the molecule is; the larger the radius of gyration is, the looser the molecule is, and it may be in a fluffy state. The Rg of the complexes was basically kept stable with the simulation, which indicated that the structure of the complexes remained stable (Fig. **3C**).

The van der Waals and electrostatic interactions between the small molecules of the complex and the protein are calculated without considering solvation, and the change of the binding force during the simulation is analyzed. Where VDW is the van der Waals force and hydrophobic interaction, ELE is the electrostatic interaction, and Binding is the sum of VDW and ELE, which can represent the binding energy of the small molecule and protein without considering solvation effects. As seen from Fig. (**3D**), VDW is more stable than ELE in the complex, and both VDW and ELE are gradually stabilized, which indicates that the binding between small molecules and proteins remains stable.

Buried SASA is a technique used to assess which parts of a molecule or molecular complex are buried inside and cannot be directly contacted by a solvent. Larger values of Buried SASA indicate stronger intermolecular interactions and larger contact regions. As seen in Fig. (**3E**), the buried SASA is basically stable, which indicates that the contact area between the small molecule and the protein remains stable, and the binding between the two remains stable. Hydrogen bonding is interaction is an important force in protein-ligand binding. Hydrogen bonds are related to electrostatic interactions and can reflect the strength of electrostatic interactions. As shown in Fig. (**3F**), the number of hydrogen bonds between small molecules and proteins mainly fluctuates between 1-3.

## 4 DISCUSSION

Currently, the main solution for DC still relies on surgical treatment, but metabolic cataracts caused by diabetic patients exhibit early onset, high morbidity, rapid progression, and insignificant postoperative visual acuity improvement, and some patients also exhibit severe postoperative complications [46-49]. As a result, an increasing number of studies have been devoted to uncovering targeted drugs, such as aldose reductase inhibitors, natural flavonoid compounds, and nanotechnology-based eye drops, with a view to ameliorating or delaying the visual effects caused by DC, which has become a pressing issue in the DC field [50-55]. In recent years, there has been a gradual increase in modern pharmacological studies of CS, which is commonly used in many areas such as hepatoprotection, immunomodulation, anticataract, and antidiabetic [56], but the feasibility and mechanism of action of CS in the treatment of DC still need to be further investigated.

The mechanism of DC has not been fully elucidated, but the activation of the polyol pathway in a hyperglycemic environment, the generation of oxidative stress, and the formation of late-phase glycosylation end-products are inextricably linked to the development of DC [52, 57-61]. In this study, the most critical target of CS for the treatment of DCs was SRC as derived from PPI analysis, and the degree value of TNF was second only to SRC, while the binding energy results calculated by molecular docking technique suggested that the binding of stigmasterol to SRC was significantly stronger than that to its binding to TNF, and that SRC played a more important role. Src-family tyrosine kinases (SFKs) play an important role in a variety of stress responses. Under oxidative stimuli, SFKs can be abnormally activated, resulting in a decrease in the expression of the catalytic subunit α1 of the lens epithelial cell Na+-K+ ATPase, which causes an increase in calcium influx and ultimately leads to a change in the homeostasis of the lens internal environment [62]. C-Src kinase is a member of the SFKs that can be activated by a variety of stimuli, and in lens epithelial cells, activation of Src kinase leads to an increase in cell migration, a weakening of intercellular junctions, and the acquisition of a mesenchymal cell phenotype by lens epithelial cells [63]. It has been shown that c-Src kinase can be activated in lens epithelial cells in a high glucose environment, which in turn induces lens epithelial cells to migrate, proliferate, and undergo epithelial-mesenchymal transition, and promotes the development of lens epithelial cell fibrosis [64]. Thus, data from several studies suggest that SRC is a key regulator in lens-associated disorders associated with the process of epithelial mesenchymal transition, which can be prompted by a high-glycemic environment, in particular, which is in line with the results of our study analysis.

The active ingredient-target network map of CS showed that stigmasterol was linked to multiple targets of action, suggesting that this component may be the main potential active ingredient for CS to exert therapeutic effects on DC. Studies have shown that as an active ingredient in many natural medicines, stigmasterol has been reported to have a variety of biological activities, such as anticancer, anti-inflammatory, antioxidant, and antidiabetic [65, 66]. Experiments have revealed that stigmasterol reduces the production of reactive oxygen species in diabetic rats as well as oxidoreductases such as glutathione reductase (GR), glutathione peroxidase (GPX), and 6-phosphate dehydrogenase, which are thought to be important in oxidative stress in cells [8, 50]. There is evidence that oxidative stress is involved in the development of DC, and hyperglycemia may lead to glycosylation of glucose, which in turn allows for the production of excess free radicals in the lens [67-69], while, stigmasterol have been reported to have strong scavenging effects on superoxide anion radicals and hydroxyl radicals [70], which is consistent with our findings. Ramu *et al.* treated a rat model of tetracycline-induced diabetes mellitus with a solution of stigmasterol, which effectively lowered fasting blood glucose and glycosylated hemoglobin levels and significantly restored the activity of gluconeogenic enzymes, such as glucose-6-phosphatase, to normal levels in rats [71]. The above studies have shown that stigmasterol has demonstrated some advantages in combating oxidative stress and controlling blood glucose levels.

Meanwhile, the enrichment results also showed the AGE-RAGE signaling pathway, which has been demonstrated to play an important role in the development of DC in a large number of studies [51, 57, 72, 73]. Non-enzymatic glycosylation of lens proteins leads to the formation of advanced glycation end-products (AGEs), accompanied by the generation of reactive oxygen species (ROS), which bind to cell surface-specific receptors for advanced glycation end-products (RAGEs) and activate NADPH oxidase (Nox) and NF-κB, serving as additional signals for the formation of intracellular ROS, which together are involved in the formation and development of DC [74, 75].

However, this study still has certain limitations. First, the network pharmacology analysis relied on existing information from public databases, and the timeliness, completeness, and accuracy of the data may be constrained by database update frequency and the quality of original studies, posing potential risks of information bias. Second, the study lacks *in vivo* and *in vitro* experimental validation, making it impossible to confirm the actual bioavailability, toxicity, and regulatory effects on cataract-related targets of the predicted active components.

## CONCLUSION

These computational findings suggest CS may exert its anti-cataract effects through multi-target modulation of diabetic metabolic pathways and SRC-mediated signaling cascades. This study not only elucidates the phyto-pharmacological basis of CS in DC management but also provides a framework for developing natural product-derived targeted therapies against diabetic ocular complications. The integration of modern genomics and computational chemistry to deconstruct the therapeutic effects of traditional Chinese herbal medicines has great clinical significance in expanding the scope of traditional Chinese medicines for the treatment of DC and promoting precision targeting.

## Figures and Tables

**Fig. (2):**
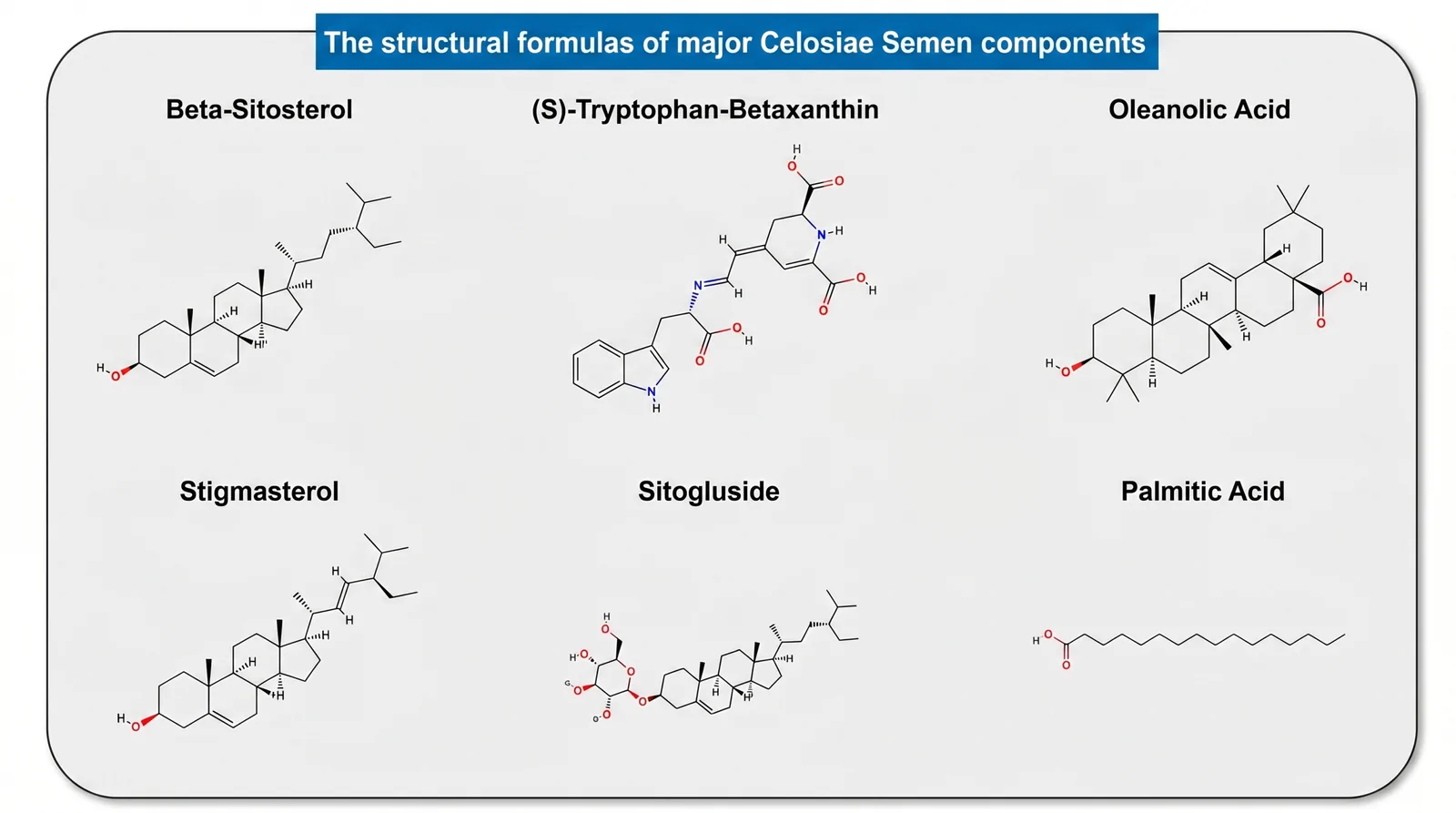
Structural formulas of major celosiae semen components.

## Data Availability

All the data and supporting information are provided within the article.

## LIST OF ABBREVIATIONS

DC: Diabetic Cataract
CS: Celosiae Semen
MD: Molecular Dynamics
OB: Bioavailability
DL: Drug-Like Properties
PPI: Protein-Protein Interaction
GO: Gene Ontology
KEGG: Kyoto Encyclopedia of the Genome
PME: Particle-mesh Ewald
BP: Biological Process
CC: Cellular Component
MF: Molecular Function
RMSD: Root Mean Square Deviation
Rg: Radius of Gyration
SASA: Solvent Accessible Surface Area
SFKs: Src-Family Tyrosine Kinases
GR: Glutathione Reductase
GPX: Glutathione Peroxidase
AGEs: Advanced Glycation End-products
ROS: Reactive Oxygen Species
RAGEs: Receptors for Advanced Glycation End-products
Nox: NADPH Oxidase
